# Collectanea

**Published:** 1827-06

**Authors:** 


### COLLECTANEA.

Floriferis ut apes in saltibus omnia libant,
Omnia nos, itidem, depascimur aurea dicta.

### ANATOMY.

*Some Directions for making and keeping Morbid Anatomical Preparations in hot Climates.* By John Davy, M.D. F.R.S. (From the Edinburgh Medical and Surgical Journal.)

It is too generally supposed that the making and keeping of anatomical preparations in warm climates is almost impossible, or attended with so much difficulty as to be practically impossible, with the ordinary means within the reach of medical officers.

This is a very mistaken notion. The changes which animal matter undergoes at a temperature between 80° and 90° Fahrenheit, (the average maximum of the highest temperature in the hottest seasons, even in intertropical climates,) do not differ in *kind* from those which occur at a temperature between 45° and 55°1′, and *a fortiori* between 55° and 70°, which may be considered the average temperature of the winter and summer seasons in Great Britain. The difference, then, in the changes is chiefly in *degree:* in a hot climate they take place more rapidly than in a temperate one,—twice or thrice as rapidly, according to the elevation of temperature. This should always be kept in mind as a maxim and principle; and, to insure success in making anatomical preparations, the rapidity of change of animal matter must be met with proportional quickness and energy of the conservative processes of art opposed to the destructive ones of nature. With the same view, and against the same tendency to change and decompose, besides quickness, great neatness and cleanliness are requisite.

Every dissection should be conducted in a regular and scientific manner, according to a certain method, and with definite objects in view. The principal objects of all dissection are three:—the detection of the effects of the disease, and the cause of death; the removal of diseased parts for preservation; and the acquisition of general anatomical knowledge. Neatness, and cleanliness, and method, conduce equally to these objects. Attending to them, obscurity, confusion, and error are avoided; the pursuit loses as much as possible its disgusting aspect; it gives information of a satisfactory kind; excites interest powerfully; and, zealously pursued, becomes almost fascinating. Farther, when the dissection is conducted on these principles, it is the source of much valuable instruction. It makes the hand dextrous for surgical operations; it produces caution in deciding upon post-mortem appearances, which are so often deceptive; and habituates the eye to the nice discrimination of what is sound in structure, and what is diseased.

When any morbid appearance presents itself, the part displaying it should be carefully examined before it is removed; its situation should be noticed, and its connexions traced. If it is considered worthy of being preserved, with that intention it should be dissected out, so as to appear to the best advantage; to require as little explanation as possible; and to be, by itself, as intelligible as possible.

If dissected out neatly and cleanly at once, free from extraneous, adipose, cellular, and muscular substance, &c. much trouble will be spared and time saved. Generally speaking, indeed, the suggestion just given cannot be too strongly inculcated. For the morbid part to become a good preparation, it should be put out of hand at once; and nothing should be left that ought and can be removed by the knife and scissors. Delay breeds neglect and forgetfulness; the nicer peculiarities of the diseased part are forgotten; after a time it ceases to excite interest; as a confused mass, putrefying or bordering on putrefaction, it is a loathsome and worthless object; and thus, in consequence of not having been finished at once, it ends in being thrown away; and an aversion, rather than a fondness, is acquired towards pathological anatomy.

The present remarks are applicable to all kinds of anatomical preparations, but more especially to those which it is intended to keep in spirits, which experience has proved to be better adapted to the preservation of moist preparations than any other liquid yet tried.

The method of proceeding in preserving preparations must, to a certain extent, be modified agreeably to the nature of the morbid parts, and agreeably to the intention of the anatomist.

If the diseased part is small, and it is wished to preserve its colour, as a portion of inflamed and ulcerated stomach or intestine, it should be immersed immediately in strong spirit, and instantly put up as it is intended it should remain. After a month, the spirit may be changed for fresh spirit, and the mouth of the vessel should be firmly secured. The blood in the part will thus be coagulated and preserved; the shape will be retained without unseemly distortions, which, when once rigid, are not easily removed; and the preparation is fit for the shelf of the museum without any farther trouble.

Preparations of the brain, spinal marrow, and nerves, should be treated in the same manner; and so treated they are most easily kept.

Thus also should be managed preparations of the eye, pleuræ, peritoneum, testes, and their tunics; and, in fact, all such parts as are liable to be injured by maceration in water and incipient putrefaction.

On the contrary, parts containing much blood, as the liver, kidneys, lungs, heart,—or stained and discoloured with blood or bile, &c. as the blood-vessels and gall-bladders, &c.—or smeared with a lubricating fluid, as the aspera arteria, primæ viæ, synovial membranes, &c. should be allowed to macerate in water till fit to be removed into spirits: of course, the hotter the weather so much the shorter must be the time of maceration, and so much more frequently must the water be changed,—as often as daily, or twice or thrice daily. And, if the part be bulky and much gorged with blood, to insure success, to prevent putrefaction during the period of maceration, either a mixture of equal parts of proof spirit and water, or saturated brine, should be used in place of water alone.

If the latter (the brine) is to be employed, it should be in readiness before the dissection is commenced, and the part should be immersed in it as soon as it is taken from the body; farther, it should be exposed as short a time as possible to the air, and should not be washed before it is put into the brine.

With these precautions, the part may be macerated till all the soluble matter is removed, (changing the brine twice or thrice,) and it is rendered fit for keeping either in spirit or in brine. If the former is preferred, it should be formed of seventy parts rectified spirit, and thirty of water; if the latter, the brine should be saturated with common salt. The proportion of rectified spirit just mentioned is found to answer best for preserving preparations in general; and it is hardly necessary to add, that, unless the brine is saturated, it will fail of the desired effect.

For the useful purposes of a museum, it is necessary that the part to be kept should be not only carefully and neatly dissected out, but also carefully and neatly put up, and that immediately, and as it is intended it should appear on the shelf. If this be neglected at the moment, the season for doing it in perfection is lost. A preparation crammed into a bottle just large enough to hold it, or thrown into spirits in a large vessel, as has been too often done without attention to suspending it in a natural way, that it may be properly seen, becomes (unless it be some very simple structure) misshapen, distorted, and confused. A preparation in such a state no skill can afterwards amend; as is very well known to those who have had occasion to attempt the annoying task of endeavouring "to make something" of a preparation, perhaps highly interesting in itself, which has been thus neglected in the first instance.

Many, perhaps, will think that the measure just recommended is not easily carried into effect; that, to accomplish it, much art and skill are requisite; and that glass vessels in plenty, and of various sizes, are indispensable.

This, fortunately, is not the case: only very moderate skill is requisite, such as every medical man ought to possess, and must possess, if he is fond of his profession, and only tolerably zealous in the pursuit of it. And, instead of many glass vessels, one or two are amply sufficient for holding all the preparations a professional man is likely to be able to collect in one year in the course of his ordinary practice.

A glass vessel of the capacity of a gallon, with a large mouth, closed either with cork and bladder or a glass stopper, is very convenient for the object in question; and it may be desirable for two of them to be constantly provided, —one for the purposes of maceration, the other for those of preservation. The preparation, neatly dissected, may be advantageously attached by a thread to a piece of cork that will float on the surface of the spirit, and keep the preparation properly suspended; or, if the preparation is lighter than spirit, as a portion of lung containing air, the same object may be effected by fastening it to a bit of lead.

In this way a great many preparations may be introduced into the same vessel: indeed, the vessel may be almost filled with them without detriment, provided each is free, and not pressed against by another, which is easily managed by using threads of different lengths. This method, it may be added, is particularly well adapted for sending preparations to England, on account of its economy, the little space required, and its security. Using it, there is no danger of the preparations being left dry and ruined by the capillary action of threads (having their ends out of the bottle) sucking up the spirit and draining the vessel: nor is there any danger of atmospheric air finding admission, provided the glass stopple or cork with which the bottle is closed is covered with moist bladder, firmly tied down, and smeared with oil when dry. Here a caution may be given, that, when bladder is thus used, the bottles should be placed out of the reach of rats, mice, and cockroaches,—

animals very fond of this membrane, and who attack it whenever it comes in their way.

Numbers written with a lead pencil on slips of paper, parchment, wood, or even the cork itself, may be introduced with the preparations when they are numerous, and there is any apprehensions of mistake; which numbers will, of course, have reference to a descriptive list that should accompany the preparations to England.

Relative to the making of dry preparations in hot climates, it will be sufficient to offer a very few observations. The unexperienced in these climates may fancy the task in question exceedingly easy, from a common and erroneous association of the ideas of proximity to the sun and parching heat. They will find it, however, a more difficult labour than can be imagined *a priori;* and for this reason, that the connexion of heat and dryness just now alluded to is, in most hot climates, of rarer occurrence than the association of high temperature with a great degree of humidity. This latter happens when the wind sweeps on its way over a great extent of sea, and on its passage becomes loaded with moisture, as is the case with the S.W. monsoon along the coast of India, the S.E. or sirocco in the Mediterranean, and the sea-breeze in the West India Islands. During the prevalence of these winds, it is very difficult to dry any anatomical preparation, and impossible, indeed, unless recourse is had to some helping circumstance, as exposure to the direct rays of the sun, or the dry heat of a charcoal fire. On the contrary, when the atmospheric heat is accompanied with dryness, as it always is when the wind comes over an extensive tract of country, such as the land-wind in India, the N.W. wind in the Ionian Islands, the S.E. on the western shore of Southern Africa, then the making of dry preparations is most easy: exposure to the wind is by itself sufficient. When dry, in every instance, the preparations should be varnished to defend them from the action of the atmosphere, and from the effect of vicissitudes in point of humidity; and then they should be carefully packed up in dried paper in a box of tight construction, to be sent home by the first opportunity.

As dry preparations of morbid parts, with the exception of bones, are of comparatively little value, nothing that is particularly interesting, capable of being kept in spirits, should be preserved in any other way.

It is not considered necessary to give any hints respecting the methods of making injected preparations. Those who have sufficient zeal for anatomical pursuits to engage in this undertaking, will have no difficulty in carrying it into effect wherever they may be, with the knowledge they must previously have acquired, and possessed of the same apparatus that is requisite in temperate climates.

In concluding, it may be remarked, that, simple and easy as are the means just now described for making and preserving morbid anatomical preparations in hot climates, they are quite adequate, and with ordinary care can never fail to succeed. Trial alone is requisite to produce conviction of their efficacy.

---

### PHYSIOLOGY.

*Action of Opium and its Constituents.*—AT a late meeting of the Institute Royale, M. DE BLAINVILLE made a very interesting report on M. CHARRET'S Memoir "On the Comparative Action of Opium, and its constituent Principles, on the Animal Economy."

Regarding opium as composed of meconic acid, morphine (which combines with it), of an extractive matter, and narcotin, M. Charret was anxious to compare the effects of these different substances, separated as carefully from each other as the chemistry of the present day could accomplish. In analysing carefully their action on each of the great functions of the living system, it was necessary, to render this comparison more instructive, not to confine the research to man, whether in a sound or diseased state, but to seek in less complicated machines, as in animals of different classes, what were their effects, so as the better to be able to analyse the therapeutic indications which they are capable of fulfilling. His experiments have been made—1st, on man, in health as well as in sickness, often on himself; 2d, on the mammiferæ, carnivorous as well as graminivorous animals; 3d, on birds; 4th, on reptiles; 5th, on amphibious animals; 6th, on fishes; 7th, on animals without vertebræ; 8th, on the mollusceæ, &c.

On all these kinds of animals, M. Charret has remarked that the solution of opium has an effect more or less considerable, according to the development of their nervous systems. Thus, in man he has remarked that it may act in three ways: 1st, on the encephalon, there determining a sanguineous congestion; 2d, on the cephalo rachidian centre, irritating and causing convulsions; 3d, on the contractile fibre, producing a direct sedative effect.

In the mammiferæ on whom M. C. has experimented, the phenomena of cerebral congestion were either very weak or altogether wanting, and the most decided phenomena were those of irritation or sedation. Thus, a larger quantity of opium was necessary to kill such animals than for man: thirty-six grains would prove sufficient to destroy a man, while it required two drachms to destroy a small cat or rabbit.

In birds, it acts only in two ways, either by irritation or sedation; but this is besides accompanied with profound stupor, indicating a stronger congestion in the brain. There is also an intestinal flux, or diarrhœa.

In reptiles, the amphibia, and fishes, there are none of the phenomena of stimulation followed by those of nervous sedation; for it is always with signs of sedation that death happens, at least in the two last classes: for with reptiles there are sometimes signs of excitation or convulsions.

As to all the animals without vertebræ, there are no phenomena of excitation, and sedation only can be produced; and therefore they die in that state.

M. Charret thinks that opium does not act unless absorbed, and he takes his chief proof of this from the experiments of Ruysten, which he has repeated, cutting away, with loss of substance, the pneumo-gastric nerves of a dog. The phenomena of narcotism took place as usual; and, besides, M. Barbier has seen a child at the breast show the same phenomena, from sucking a nurse who was under the influence of opiates. If the employment of opium is unfavourable to digestion, M. C. shows that this arises from paralysing the muscular coat of the stomach, suspending its function as a direct effect, and causing nausea and vomiting as secondary effects. Constipation from opium he thinks equally arises from the momentary paralysis of the muscular coat, and diminution of the mucous exhalation, in consequence of the increase of that of the skin.

Absorption appears not to be modified by the employment of opium. With regard to the circulation, it is certain that it causes a stagnation and engorgement in the capillary vessels. On nutrition, opium, if frequently used, has a fatal influence, and causes a kind of marasmus. The exhalation appears always to be augmented, either on the surface of the skin or on the surface of the intestines, or in the splanchnic cavities. As to the secretions, it appears to have no sensible effect on the production of tears or saliva, and probably not on the bile or the pancreatic juice: as to the urine, it is diminished if the perspiration is increased. The spermatic secretion is not affected.

M. Charret has occupied himself in observing what are the differences of action of the extract of opium, according to its mode of preparation, and also as to its being indigenous or exotic. In tincture, it is evident that the action of the opium is more energetic, from the facility of diffusion of the vehicle; therefore this form is preferable in cases of spasm, and to be avoided when congestion is dreaded. It has been said that indigenous opium does not act so well as the exotic: M. C. merely observes, that M. Vauquelin found the same quantity of morphine in both.

Opium injected into the rectum produces effects as rapid and intense as when thrown into the stomach. Applied to the skin, it produces, first, local phenomena, and then general, which tends to prove that its first effect is direct.

M. Charret has experimented on morphine, and finds that this substance, in the form of acetate, dissolved in acids, rubbed up with oil, or crystallised, has always very nearly the same effects; dissolved in alcohol, its action is more energetic. In man, he observes that morphine does not cause so much perspiration, and increases the urinary discharge. M. C. thinks it less efficacious than opium in intermittent fevers, and prefers it in cases where congestion is to be feared, and in phthisis where the sweats are to be repressed.

With regard to narcotine, its effects are extremely variable; but with M. C. its effects so much resembled those of the morphine, that, if he had not procured it from a good source, he would have doubted its purity.

M. C. concludes by regretting the very unsatisfactory chemical analysis that has yet been given of opium, and is inclined to think the watery extract the surest preparation. (*Archives Generales.*)

### PATHOLOGY.

*Case of Poisoning by Stramonium.* By CH. D. MEIGS, M.D.—I believe that examples of poisoning by stramonium are not very common: certainly, accounts of this affection are not so very numerous as to have become fatiguing, and, as there is some difference in the statements of symptoms produced by the use of this weed, I have related the case below. When I was student of medicine, my teacher, who was much employed in practice, took me to see a young girl, who had swallowed a number of seeds for the purpose of self-destruction. She was insensible, completely tetanic, and had a very dilated pupil. She recovered.

The effects produced by stramonium on some of the first settlers of Virginia, was a high degree of intoxication.

The case which I am going to describe occurred in a girl two years and a half old, daughter of Mr. C. Stelwagen. On the 24th October, 1824, (forenoon,) she had found a small bag, containing stramonium seeds, of which she ate an unknown quantity. The first symptom was a high degree of exhilaration, in which she excited much merriment by her extravagant gestures and speeches. This soon became alarming; and when I was called to see her, she was laughing, crying, and singing, by turns, proceeding from one to the other state with the greatest rapidity. She occasionally started with great force and alarm, crying out that she was going to fall; when she would cling to her mother with as much desperation as if she was about to be thrown from a precipice. She would next become calm, then whistle, and afterwards point with her finger at muscæ volitantes, which she followed with the eye and hand, at last clutching at them, with an appearance of disappointment at the want of success.

The colour of her face was of a scarlet red. I have certainly never seen so intense a red in scarlatina. Her skin was hot; pulse much accelerated; and tongue and fauces dry and red: the former was so dry that it glistened. The face, neck, and breast were covered with hundreds of small brilliant petechiæ, many of which had a stellated form.

After an emetic, which operated very well, and brought up only one seed, I gave her senna infusion, with repeated enemata. The nurse told me she had found forty seeds in the evacuated matters.

The cerebral symptoms I have described gradually diminished till midnight, when she fell asleep. On the 25th, she was tolerably well. The petechiæ were still quite evident, not being much changed. A troublesome itching of the whole skin, which came on yesterday, was gone.

27th.—The child is well, but the petechiæ are not gone.

Nov. 4th.—They are no longer visible. (*North American Med. and Surg. Journal.*)

### PRACTICAL MEDICINE.

*Epilepsy.*—Dr. USHER PARSONS, of Providence, relates a case of epilepsy, of an obstinate character and long standing, cured by Dr. MANSFIELD's galvanic treatment. In this case, Dr. Parsons removed the cuticle on the back of the neck by means of a blister about the size of a sixpence, and a similar portion from the inside of the knee. To the sore on the neck he applied a small silver plate, which had a small staple affixed to its lower edge, in which was fastened a conducting wire. The wire was extended down the back into a belt of chamois leather fastened around the waist; it was continued in this belt round the side, until opposite the groin, and thence down the thigh, until it reached, and was attached to, a zinc plate placed over the sore on the knee. Dr. Mansfield's directions are to apply to the sore a small bit of sponge moistened in water, and corresponding to the denuded place in the neck. Over this, a large piece, of the same size as the metallic plate, also moistened, is to be laid; and next to this the plate itself, which must be secured by adhesive straps. The wire down the back should be long enough to permit free motion of the body. The zinc plate at the knee is to be fastened in the same manner, with the exception that, for the second layer of sponge, a layer of muscle must be substituted. The apparatus will continue in gentle action from twelve to twenty-four hours; when the sores require cleansing, as well as the plates. Dr. Parsons applied this apparatus to his patient, who discontinued its use after a perseverance in it of six months; and, although two years have elapsed, he has not had an epileptic paroxysm since the date of its first application. (*New England Journal of Medicine and Surgery.*)

*Asthma.*—Dr. Chiarenti having often observed the good effects of sudden exposure to the fresh air, particularly with the face opposed to the wind, in paroxysms of asthma, tried, during a paroxysm of this disease, to which he himself is subject, to introduce the tube of a pair of bellows into his mouth, and to blow with force a great quantity of air into the lungs. The event justified his attempt, and by the aid of this simple operation he can, in a very short time, overcome the most violent attacks of asthma. After repeatedly trying this experiment on himself, he tried it on others, and with the same success. Dr. Chiarenti, from the numerous facts he has collected on the subject, believes he may confidently assert that this insufflation of air into the lungs is not only capable of instantly arresting a paroxysm, but even of radically curing the disease, provided it is not the result of organic alterations!! (*Annali Universali di Medicina.*)

*Amaurosis cured by Tartar Emetic.*—A woman, named Baldini, ætatis thirty, when ten years old was, after a prolonged fever, seized with paralytic trembling all over the body. Two months after, severe pain came on in the orbit of the right eye; the paralytic tremblings ceased, and were succeeded by complete amaurosis of the right eye. The pain of the orbit, and parts around it, continued till she was twenty-one years old, when an abundant mucous discharge from the nostrils came on, and the pains ceased. In the month of November, 1825, the pain returned, and fixed in the left eye, becoming extremely violent. The faculty of sight was soon nearly lost, so that she could hardly distinguish night from day. Local and general bleedings, purgatives, and blisters, were in turn employed without success.

When this patient came into the hospital, the eye showed no external lesion; the pupil was dilated; and a similar dilatation, with insensibility of the pupil to light, existed on the right side. In the bottom of the eye, something whitish and horny looking could be perceived; there was also strabismus. The patient having refused the introduction of a seton in the nape of the neck, a vegetable infusion was prescribed, to which was added a grain of tartar emetic.

Second day.—Two grains were given, which produced vomiting.

Third day.—The patient could distinguish various objects with her left eye.

The same treatment, increasing the dose to three grains, was continued to the seventeenth day, when the left eye had quite recovered its power of vision, and with the right she could distinguish the buttons on a coat. (*Ibid.*)

*Observations on the Inexpediency of sending Consumptive Patients to Madeira.* By A. H. Renton, M.D. (From the Edinburgh Med. and Surg. Journal.)

The object of the following observations is to save from much unnecessary suffering and inconvenience a hapless class of beings, for whose relief but little can be done by medicine, and whose fate the most callous of us must necessarily deplore. I allude to those invalids sent hither from England in the last stage of pulmonary consumption.

It would be foreign to the intention of this communication, to make any remarks on the nature of the symptoms which indicate diseased lungs, or to say a word about the cure of a disease which, in its advanced stages at least, most medical men believe to be, in the present state of our knowledge, irremediable. No unusual share of acuteness is necessary to detect confirmed consumption, and still less is requisite in forming an opinion as to its result. My only object is to call the attention of my professional brethren to the inutility—I had almost said the cruelty—of the practice of annually banishing from home and its comforts a host of devoted victims, whose very hours are numbered, and who are thus, by the decision of their medical attendant, deprived of the only consolation which can be afforded them in their descent to the grave, the society and soothing attention of affectionate relatives. The increasing frequency of this practice shows that the judicious remarks of others on this subject have been neglected, and that the following observations may not be unnecessary.

That this measure is frequently adopted merely to gratify the wishes of the unfortunate sufferer, or those of his friends who are, naturally enough, anxious to leave nothing untried in their search for means of relief, is probably true. But it is evident, both from his own account, and from the written statement which he brings with him, and which but too frequently holds out the most flattering hopes of recovery to breathless, I may say *lungless*, objects, worn down to the bone by profuse colliquative discharges, that, generally speaking, the patient himself has little to say in the arrangement; and that it is principally in obedience to medical advice that he undertakes a voyage, productive of nothing but mischief and disappointment. What object a practitioner can have in view in sending invalids abroad in such hopeless circumstances, it is difficult to conceive. One would suppose that the dissection of a single body dead of phthisis, which had run its ordinary course, would be sufficient to convince the firmest believer in the certainty of the efficacy of our art, that neither change of climate, nor any known means, can avail in the repair of such extensive disorganisation, as must evidently have existed for a long time previous to the death of the subject. As to any relief from suffering which is obtained by dragging out the last few months of existence in a warmer climate, the inconvenience which is invariably experienced during the first (and of his the whole) part of a residence abroad, would more than counterbalance it, were it much more considerable than it actually is; for he must know little of his own kind who is not aware of the difference between the comfort which is to be derived from the watchful attendance of attached friends, and that to be expected from the usual, however kindly offered or well intended, civilities of a stranger. So uniform is the result of this practice, that the annual importation of invalids from England is thought a fit subject for ridicule. " La vai mais hum Inglez a Laranjeira;" —there goes another Englishman to the Orange-tree, (the Protestant burying ground,)—has become the joke among the boatmen on landing these unfortunates on the island.

The following table, limited as it is, will give a tolerably correct idea of what is to be expected from a residence here in lung cases. It is taken from those of which I happen to have memoranda, and which form a part of those which have been sent here during the course of the last eight years. It does not include the invalids who have come out this winter, many of whom will never see their native shores again.

In the cases marked Confirmed Phthisis, there were copious purulent expectoration, diarrhœa, &c. and almost all of them terminated fatally here. I examined the bodies of fifteen of them after death, in the presence of some of my professional friends; and in every instance the lungs were found almost completely disorganised. The extent to which the process of disorganisation may proceed before death, is better exemplified here than in Britain, as the patient's progress, *à son heure suprême*, is less liable to be hastened by accidental inflammatory attack. In some of them the pulmonary symptoms were stated to be merely secondary, and the liver was denounced as the offender in chief; but in only one instance (that of a gentleman from Scotland, in whom that organ was found enormously enlarged, and of which there was sufficient evidence before death,) was there found the slightest deviation from healthy structure in any of the other cavities. From this I exclude intestinal ulcerations, which are so generally met with whenever the disease has had an opportunity of running its victim completely down.

Some of those marked Incipient Phthisis, were probably not fully entitled to an appellation so ominous. Their general character was young people who were said to have "overgrown themselves," and who had been subject in England to inflammatory attacks, having cough, &c. Others had suffered from neglected or mistreated inflammation, and in many there was a strong family predisposition to pulmonary disease. Most of them, I have little doubt, would now have been in their graves but for the precautionary measure which was adopted.

The other diseases were asthma, scrofulous glandular enlargements, and rheumatism, all of which were benefited by a residence here.

| | | |
|---|---|---|
| Cases of Confirmed Phthisis | | 47 |
| Of these, there died here within six months after their arrival | 32 | |
| went home in summer, and returned and died | 6 | |
| left the island, but of whose death we have heard | 6 | |
| and not since heard of (probably dead) | 3 | |
| Case of Incipient Phthisis | | 35 |
| Of these, there left the island, much improved in health, and of whom we have had good accounts | 26 | |
| also improved, but not since heard of | 5 | |
| and have since died | 4 | |
| Other diseases | | 15 |
| | | 97 |

From this it appears that there are in England two sorts of cases in which transportation is recommended,—those which are curable, and (principally) those which are not. Regarding the latter order, I shall merely observe that, in cases of the common tubercular phthisis, in which suppuration has commenced, a prolongation of existence, (and that under severe restrictions,) is all that a residence here can be expected to afford. When it has proceeded to any considerable extent, I should consider it the duty of a medical attendant not only not to advise the adoption of such a measure, but most earnestly to dissuade from it those who, from hearsay evidence of the recovery of others in circumstances similar to their own, may feel disposed to fly to it as a last resource.

That great and lasting benefit is to be derived from even a temporary residence in this climate, which is probably inferior to no other in cases where pulmonary disease is merely threatened, or where strong family predisposition to it exists, many living examples sufficiently prove. But, even under such comparatively favourable circumstances, it ought to be strongly impressed on the mind of the invalid, that half measures are worse than useless; and that no advantage is to be derived from climate, however fine, unless it be steadily seconded by the utmost caution and prudence on his part.

---

### SURGERY.

*Observations on Laryngotomy and Tracheotomy.* (From a Clinical Lecture delivered to the Students of Surgery in the Royal Infirmary of Edinburgh, by GEORGE BALLINGALL, M.D. F.R.S.E. &c.)

On going round the ward on the 10th of January, this man (John Yule) was reported to me as suffering from sore-throat, but did not attract my particular attention, nor did I observe any thing about him calculated to give me alarm. On the evening, however, of that day, he became affected with marked symptoms of laryngitis. Dr. Lubbock, the house-surgeon, very promptly abstracted forty ounces of blood, administered an antimonial solution, and ordered the application of leeches to the larynx. By these means the disease seemed for a time to have been arrested. The patient's symptoms were alleviated; and, on my visiting him about ten P.M., he expressed himself much easier, breathed with less effort, and with less of that stridulous noise characteristic of the complaint under which he laboured. When I visited him again on the following morning, his symptoms had become very alarming; his pulse 120, and weak; his voice enfeebled; his respiration laborious, and his countenance livid. After a moment's consultation with my colleague Dr. Campbell, who accompanied me, I proceeded to open the windpipe, making a crucial incision down through the cricoid cartilage, and inserting a silver tube into the trachea: the patient, who at this moment had ceased to breathe, and was thought to be dead, began again to respire; his pulse, which was for some moments imperceptible, became again distinguishable at the wrist. He swallowed some brandy and water, and survived till three in the afternoon.

* * * In speaking of the proper points for perforating the windpipe, I adverted to the division of this operation into laryngotomy and tracheotomy, and endeavoured to point out the cases in which the one or the other was the preferable operation. In cases like the present, where the patient runs a risk of suffocation from œdematous swelling of the rima glottidis, and where there is no reason to believe that the inflammation extends further down, it may be sufficient to make the perforation where I did. And this is the situation where, of all others, from the thinness of the superincumbent parts, it may be most readily accomplished. The operation has been represented as one which may be as easily performed as bleeding in the arm; and in a case of this kind it certainly may be so, where the obstruction is entirely within, and no swelling of the neck without: but you had a very recent opportunity of seeing how much circumstances are altered in some particular cases.

On the evening of the 5th of February, a female patient of Dr. Spens's was reported to me as labouring under cynanche parotidea; and, while in the middle of my clinical lecture, one of the house surgeons came in and reported to me that this patient was so much worse as to lead him to apprehend immediate suffocation. On visiting her along with you, I found her affected with a very large swelling on the left side of the jaw, involving the parotid, the submaxillary glands, and the tonsils, and extending down over the neck. Her respiration was extremely difficult, and her face turgid. I was told that she had been admitted with fever about three weeks before, and that this swelling having recently supervened, it had partially suppurated on the surface, and had been opened. This opening I proceeded, in the first place, to enlarge, thinking that by this means I might give vent to matter, and relieve the urgent symptoms. This, however, gave no relief, and produced no diminution of the internal swelling; which, upon introducing my finger into the mouth, I found to extend across the fauces, covering the aperture of the glottis, and thus producing a mechanical obstruction to the admission of the air. Although I considered the case almost desperate, and mentioned to you at the time that she might possibly not survive half an hour, yet, as the patient was comparatively a young woman, and her pulse pretty firm, I considered it my duty to obviate the risk of suffocation by making an opening into the windpipe. As the obstruction in this case was not from any thing within the tube, but from the tumor covering its orifice, it was obvious that an opening into any part of it below that orifice would answer my purpose; and I made a longitudinal incision along the fore part of the larynx, with a view of perforating the crico-thyroid membrane. From the turgid state of all the vessels in the neck, a considerable hemorrhage took place, not from any vessel of sufficient magnitude to be tied, but a general oozing, particularly from the upper and lower extremities of the wound, in such quantity as to induce me to defer opening the trachea until it should be suppressed. The hemorrhage from the lower angle of the wound was a matter of indifference, because, by putting the patient in an erect posture, and perforating above the bleeding point, the entrance of the blood into the trachea could be easily prevented; but the admission of blood into the tube from the upper point of the incision was not so easily obviated. I therefore attemped to restrain the bleeding by pinching up a portion of the cellular substance, and surrounding it with a ligature; but, this not proving sufficient, I applied a piece of caustic to the bleeding point, and partly by this means, partly by exposure to the air, the hemorrhage was in a few minutes suppressed; when I completed the operation by slitting open a portion of the larynx, and introducing a silver tube. This was in some measure done at random, from the parts not being distinctly marked, and from my being afraid of renewing the hemorrhage by dissecting them more completely. The patient, as was to be feared, sunk rapidly, and died in about twenty minutes after the operation. The parts were exhibited to you at the following lecture, and are now deposited in the Museum of the Royal College of Surgeons. The aperture was found, as I told you it would be, in the lower part of the thyroid cartilage, between its alæ, and extending downwards to the crico-thyroid membrane.

Now, gentlemen, what I wish you particularly to observe is, the remarkable contrast between these two cases. In the former, the patient was a robust male, advanced in life; the neck unincumbered with fat, and free from any adventitious swelling; the pomum Adami prominent; the cartilages hard; and the different parts of the larynx so well marked, that they might have been demonstrated through the superincumbent integuments. In the other case, the patient was a female, comparatively young, the neck swollen, and the blood-vessels turgid; the larynx not prominent, nor its cartilages hard. No two cases could give a better illustration of the different circumstances attending the performance of this particular operation; and no two cases could better illustrate the general advantages of clinical observation. In the one case, there was scarcely as much blood lost as would soil one's fingers: in the other, a hemorrhage, which although of no great amount, yet it became an important object to suppress; for, had any part of the blood got into the trachea in this patient's enfeebled state, it would, I apprehend, have produced immediate suffocation,—the very occurrence which I was called upon to prevent. In the one case, the operation was as simple, smooth, and easy, as ever it was described in a system of surgery; in the other, it was such as you must sometimes expect to meet with in the realities of life.

With these two cases of bronchotomy, those gentlemen who were here in the early part of the season had an opportunity of comparing that of Betty Oswald, a patient of mine, who was admitted into the hospital immediately after having been delivered of a natural child, and having made an attempt at suicide, in which she had divided the lower part of the larynx by a ragged unequal wound. This wound was, I may say, completely closed, and no air passing through it, when the patient was seized with inflammatory symptoms in the trachea, stridulous breathing, and sense of impending suffocation. These symptoms were relieved by making a perpendicular incision into the larynx, from the site of the original wound down through the cricoid cartilage. The aperture thus made was again diminished to a very small extent, when a recurrence of the urgent symptoms induced the house surgeon again to enlarge the wound; and this, aided by general and local bleeding, succeeded in rescuing the patient from the very alarming state in which she was for some days. In this case it appeared that some impediment had been formed to the restoration of the natural channel for breathing by the aperture of the glottis, perhaps in consequence of some deposition of lymph from the severe inflammatory attacks which she had sustained. All attempts to close the wound in the larynx were now abandoned, and the patient became habituated to the use of a silver tube in the wound, through which she breathed with tolerable comfort, and in this state she left the hospital.

*Wound of the Radial Artery; with Observations on Hemorrhage.* (From a Clinical Lecture, &c. by Dr. GEORGE BALLINGALL.)

The next case which I shall notice is that of Thomas Sutherland, who was admitted into hospital on the 6th January, having received a small wound from a piece of bottle-glass, opening the radial artery at the point where it comes out from beneath the extensor tendons of the thumb, and passes between the metacarpal bones of the thumb and fore-finger towards the palm of the hand. The house surgeon, finding it impossible to secure the artery in the wound, had passed a ligature upon it immediately above the carpus, at the same time introducing a piece of sponge into the wound. On the evening of the 9th, bleeding occurred from the original wound, preceded by pain and swelling in the fore-arm, with some degree of symptomatic fever; for which leeches had been applied to the arm, and a poultice to the hand. The hemorrhage was again suppressed by the sponge, and nothing unfavourable occurred until the evening of the 11th, when hemorrhage took place from the radial artery, where it had been tied above the wrist. I immediately enlarged the wound upwards and downwards, and passed two ligatures around the artery, one above, and the other below the bleeding point. Hemorrhage recurred two or three times from the original wound on the 14th, and was easily restrained by moderate pressure.

On the morning of the 15th, I was called to this patient in consequence of another hemorrhage from the primary wound. This had ceased before I reached the hospital; but, upon examining the hand, I found it swollen and tense, with a collection of purulent matter under the integuments of the metacarpus. This was discharged by an incision of about an inch and a half in length across the back of the hand; and between two and three o'clock of the same day I was called away from my class in the College, in consequence of a recurrence of the hemorrhage from this man's hand. I now extended the original wound upwards and downwards, and made an attempt to secure the bleeding vessel; but this I found impossible, on account of the ulcerated, or rather sloughy, state of the parts, in consequence of which the ligature would not keep its hold. I ascertained, however, (upon this, the first occasion on which I had myself seen the hemorrhage from this wound,) that the blood came distinctly from the superior extremity of the wounded artery, and that it was not in any degree commanded by pressure on the ulnar, proving that it was not supplied, as I had hitherto supposed, by the inosculations between the radial and ulnar arteries in the palm of the hand. I therefore directed one of the dressers to restrain the hemorrhage by pressing a piece of sponge upon the bleeding orifice, and requested my colleagues to meet me at six o'clock, after the clinical lecture. I then proceeded, with their approbation, to tie the humeral artery a little below the middle of the arm, and from this time all further hemorrhage ceased. The ligatures upon the radial artery separated in a few days, and that upon the brachial on the fifteenth day after its application; the wounds healed kindly, although slowly; and the man has been recently discharged, with the motions of his arm unimpaired.

In speaking of this case, I illustrated it by a reference to numerous others in the writings of Gooch, O'Halleran, White, Hodgson, Guthrie, and Turner. I observed that this case, however troublesome it had proved, was by no means singular, nor even very rare. I showed you that, in one case quoted by Gooch, a young man lost his life in consequence of a wound of this artery; and that in another case, related by O'Halleran, after repeated hemorrhages for a month, the limb was amputated. I find also that the frequency of secondary hemorrhage from this artery was a circumstance which did not elude the comprehensive grasp of John Hunter's mighty mind. In a manuscript copy of notes from his lectures, in Dr. Knox's possession, Mr. Hunter mentions the radial and ulnar arteries as those, of all others, most prone to secondary hemorrhage from ulceration of their coats; and my colleague, Dr. Campbell, has called my attention to a peculiarity in the situation of these two arteries, which is well worth noticing in any attempt to account for this. You will recollect that these vessels run for a considerable space on the lower part of the fore-arm, covered only by the common integuments above, and supported beneath by parts almost purely tendinous: the former circumstance rendering them more liable to injury; and the latter, perhaps, leading to their more frequent ulceration, in consequence of a less intimate and vital connexion with the contiguous parts. But, to return to the fact, and to the particulars of Sutherland's case, you will recollect that I pointed out, on one of Rosenmuller's plates, the inosculations by which I conceived the repeated hemorrhages in this instance to have been supplied, through the medium of what my late distinguished master, Dr. Barclay, has termed the ancono-carpal arch, formed by the anastamoses of the extreme branches of the interosseal with the radial and ulnar arteries on the back part of the carpus.

This case forces strongly upon our minds the singular revolution which has taken place in the opinions of surgeons relative to the ligature of arteries within the last sixty years. *They* were formerly afraid to tie any considerable artery, for fear of mortification of the limb: *we* are now apprehensive that the ligature of a main trunk may prove insufficient to suppress a hemorrhage from one of its minor branches. "When the brachial or femoral artery is wounded, though the patient should not perish by the hemorrhage, the limb must soon die for want of nourishment." This was the language of Gooch in 1767. "When circumstances tending to prevent the establishment of a collateral circulation do not exist, we need not apprehend the death of any part in consequence of a deficient supply of blood after the ligature of its main artery." This was the language of Hodgson in 1815; and if you look into the accurate and instructive work of this author, you will find a case in which the ligature of the brachial was found insufficient to suppress a hemorrhage from this same unlucky radial artery; the wounds of which, I may safely assert, have given more trouble to surgeons, and proved more disastrous to patients, than those of any other artery of a corresponding magnitude in the system. Surgeons have sometimes been made to pay in purse, as well as in person, for the mismanagement of such cases. Of this I recollect a remarkable instance which occurred in a provincial town in England, not many years ago, where a surgeon was prosecuted, and cast in damages, at the suit of a cooper, who had lost the use of his arm, and was disabled from following his trade, in consequence of the tight bandaging employed to restrain a hemorrhage from the radial artery. This case was strongly impressed upon my recollection by a very extraordinary coincidence: while reading the newspaper report of it in the mess-room at Nottingham, where I was then quartered with the 33d regiment, I was called to the assistance of a soldier who had accidentally wounded his radial artery in whetting his razor; and, having the fear of tight bandages fully before my eyes, I of course proceeded to secure the wounded vessel with ligatures.

*Removal of the Os Astragalus.*—Dr. ALEXANDER H. STEVENS, professor of surgery in New York, removed, last summer, the astragalus, after a compound luxation of the ankle-joint, otherwise irreducible. The man has recovered, with very trifling deformity of the foot, and with a *flexible joint.* He walks with very slight lameness. *(North American Med. and Surg. Journal.)*

---

### MISCELLANEOUS.

*Effects of Green Tea.* By W. NEWNHAM, Esq.—During the spring and summer of the year 1822, I was attacked by increased arterial action of the cerebral vessels, for which I was twice bled largely, besides repeated leeching, and the uniform pursuit of a strictly antiphlogistic system of diet and medicine. But the distressing headache, throbbing of the carotid arteries, &c. were only palliated, not dissipated, by these means. Nor is this wonderful; for the malady had been slowly creeping upon me for a very long time, and had only been roused into the state of acute suffering by a course of intemperate study. Having often formerly found relief from taking green tea for less severe headache, I determined upon giving it a trial. The pain was very intense when I first employed the remedy, and I never shall forget its effects. It was a strong infusion, and very soon after I had swallowed it the severity of the pain was diminished, and a delightful calm stole upon me. This was accompanied with considerable exhilaration of spirits; but I am unable to say if this arose from the pleasing consciousness of diminished suffering, and of being in possession of an agent capable of controlling the excessive action of the cerebral vessels, or from the immediate effect upon the brain of the agent itself.

This quiet calm speedily gave way to a variety of very painful sensations,— an almost insupportable anxiety about the præcordia, palpitation and fluttering of the heart, general tremor, and a peculiar distress, which I have always found it impossible to define or describe by words. During all this time my spirits did not forsake me; for I felt that my head was better, and I had suffered so much and so long from the state of that organ, that to obtain a prospect of relief was a source of unspeakable pleasure. My friends saw , and were alarmed by the effect of the tea; but I assured them I was better, and that I would, on no account, exchange my present distress for the dominion of my old enemy, headache. The symptoms of agitation gradually subsided, and I slept that night with a greater degree of quiet than before. During the progress of my illness, I again had recourse daily, for some time, to the same remedy, and always with the effect of quieting the excitement, as well as with the recurrence of the same symptoms of anxiety and oppression, but in a minor degree of intensity. It appeared to me that its first and immediate influence was exerted on the brain, and that the centre of the vascular system was affected secondarily, and as a consequence of such influence. The circumstances related will, however, clearly prove that the agency of this infusion, in arresting cerebral excitement, is very considerable, and that it is well deserving the attention of the profession.

This sketch would scarcely be complete, unless I were to state the effect of green tea upon myself subsequent to my restoration to health. Having now for some years lost all traces of my former headaches, there has been no brainular irritation to subdue, consequently the remedy has been taken rather as a luxury than a medicine, and only at my usual hour of drinking tea, about nine o'clock. When this has been the case under common circumstances, it has generally happened that slight symptoms of præcordial anxiety, and a very wakeful night, have followed: but, if the brain shall have been more than ordinarily excited by any animated conversation during the evening, or by late and close attention to connected thought, the uneasiness about the heart has vanished, and the sleep has been most refreshing.

Thus satisfied of the salutary influence of green tea upon a peculiar morbid action of the cerebral system, I felt anxious to inquire what would be its effect during a state of health, and I determined on submitting myself and my pupils, Mr. Carter and Mr. Nichols, to its influence. I had taken care that the latter gentlemen should not be informed of the effects which might be expected from the experiment, in order that I might secure an unbiassed report of their sensations.

An ounce of the very best gunpowder tea was infused in a pint of boiling water for twenty minutes, and divided into three portions, of which each took one. On myself, the symptoms produced were precisely such as have been before described, except that, as there was no disease to combat, so, consequently, the delightful sensation arising from the control of such malady was wanting. Hence I experienced all the anxiety and oppression, with less of the pleasurable feeling; but I could not mistake the old effects, although they were not so violent in degree. My pulse, which before taking the tea was perfectly regular at eighty strokes in the minute, was at first quickened and rendered fuller, but in fifteen minutes it had again fallen to eighty, and had become very irregular and intermitting; in half an hour it had fallen to seventy-six, and continued exceedingly irregular. A feeling of anxiety oppressed the heart, and a general tremor had come on, and remained for some hours, and indeed, to a certain extent, for the remainder of the day. The same experiment, repeated ten days afterwards, was attended with precisely similar results.

On Mr. Carter, the immediate effect upon the pulse was that of quickening it; but it afterwards fell below its natural standard, and became irregular and intermitting. The sensations he described to me were those of temporary exaltation; "he felt a greater degree of confidence in himself;" which, however, quickly gave way to oppression and anxiety about the heart, palpitation, a slight degree of nausea, general tremor, and a feeling of debility, as if his knees refused to do their office in supporting the body;—all circumstances which, as an independent and unsophisticated testimony, go far towards confirming the position before laid down, that the agency of green tea is exerted principally on the nervous system, and through it upon the several functions of the animal machine.

The influence of this medicine in controlling the inordinate excitement of the vessels of the brain, was even more particularly elucidated in the case of Mr. Nichols. Without being aware that it might have any effect upon the experiment, this gentleman had taken for his lunch, about an hour previously, some Edinburgh ale. His pulse was at ninety-two, with a hurried, vibrating action; but, shortly after taking the tea, it was reduced to eighty-four, and even eighty, while his feelings were those only of increased comfort. Disposed to smile at his fellow pupil's uneasiness, and to ascribe it to peculiarity of constitution, he came to a repetition of the experiment with great pleasure. But on this occasion no potation of Edinburgh ale had preceded that of the tea: his pulse was, as before, considerably reduced in frequency, and became very feeble and fluttering; while his sensations of uneasiness about the heart, general tremor, and debility, were not a little distressing to him for some hours, and scarcely even completely subsided during the remainder of the day.

The sequel of my pupil's history is too characteristically illustrative of the mutual action and reaction of green tea and alcoholic stimuli, to be omitted. It so chanced that, during a few months' residence with me last summer, I was in the frequent habit of taking green tea in the evening. Upon these occasions, he invariably complained next morning of a wretched, sleepless, and miserable night. I took this opportunity of watching the influence of wine and strong beer upon this state of nervous irritability; and, whenever either of these were taken after a cup of green tea, the wakefulness and general distress were not present. (From *Some Observations on the Medicinal and Dietetic Properties of Green Tea.)*

*Vaccination.*—At a late meeting of the Academie de Medicine, M. P. Dubois read the Annual Report of the Commission for Vaccination in France for 1825. This report contains, among other remarkable things, a discussion on the fact that M. Kergaradec had communicated from M. Guillon de St. Pol-de-Leon, at a former meeting. It will be recollected that this physician asserted that he had produced the true vaccine influence with the matter taken from a varioloid patient; and, consequently, that this matter and that of the vaccine virus were the same. The commission in their opinion express doubts of the reality of the results that M. Guillon says he obtained. They cannot be persuaded that varioloid matter can produce vaccination, nor that there can be any identity between the diseases. But supposing it to be true, there are two reasons which should prevent any use being made of the discovery: the one is, that the varioloid is not easily distinguished from true small-pox, except towards the end; it would therefore be to be feared that most practitioners would be deceived, and communicate the small-pox instead of the varioloid or vaccine. The second reason is, that the efficacy of the vaccine virus being proved by thousands of proofs, it is unnecessary to seek a new antidote to small pox, where we already have one sure and free from danger. *(Revue Medicale.)*

